# Supplementary material for: 3D multiscale shape analysis of nuclei and in vivo elastic stress sensors allows force inference
Source: Biophys J. 2025 Jul 22;124(17):2784–96. doi: 10.1016/j.bpj.2025.07.015 (PMC12461028; doi:10.1016/j.bpj.2025.07.015)
Supplement: Document S1. Figures S1–S5 [file mmc1.pdf]

**Biophysical Journal, Volume *124***

## Supplemental information

# 3D multiscale shape analysis of nuclei and in vivo elastic stress sensors allows force inference

**Alejandro Jurado, Jonas Isensee, Arne Hofemeier, Lea Johanna Krüger, Raphael Wittkowski, Ramin Golestanian, Philip Bittihn, and Timo Betz**

# Supplementary Figures

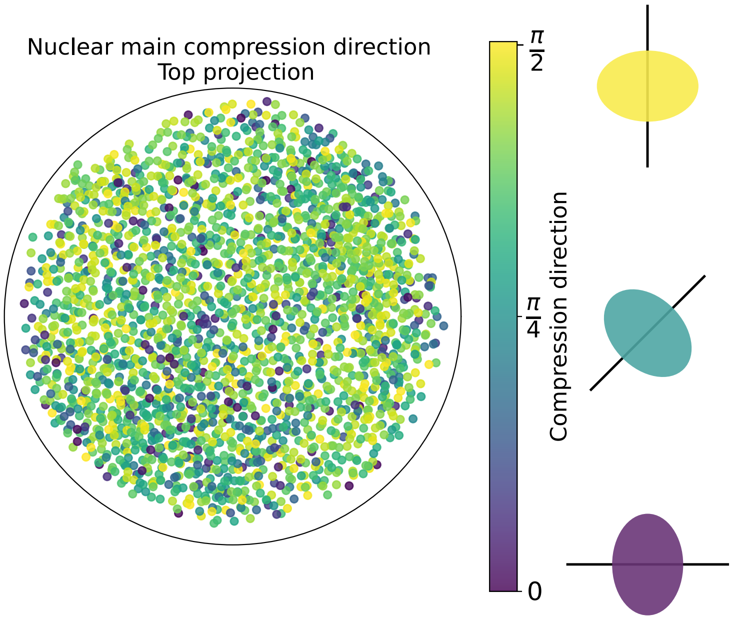


Figure S1: Top view of a 5 hpf Zebrafish with color-coded nuclear orientation. The orientation of maximum nuclear compression was calculated in Zebrafish, as well as in reconstituted muscle tissue. In the former, no particular patterns of deformation were observed, either spatial or temporal.

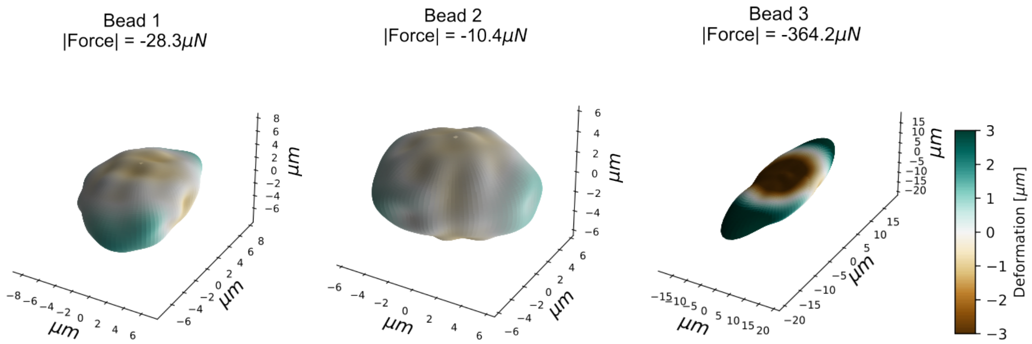


Figure S2: Three PAA beads belonging to the 5hpf Zebrafish dataset. The "outlier" Bead 3 belongs to a very deformed detection, with radial displacements > 3µm, which resulted in measured compression forces above 300µN.

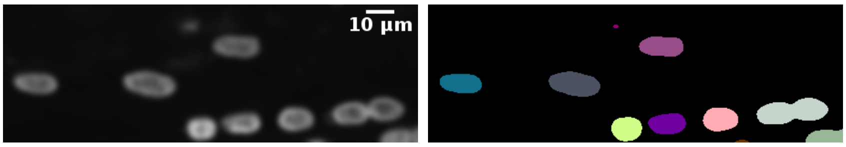


Figure S3: Segmentation failure for multi-nucleated cells in reconstituted muscle tissue. Nuclei in very close proximity (bottom right structure) are difficult to separate, and are recognized as a single, elongated structure, which created a small sub-population of detections with high "deformation" values in our study of skeletal muscle tissue. The intrinsic scale separation during the Spherical Harmonic decomposition of the detected objects allows an easy filtering of faulty segmentations. In this case, objects with a base coefficient $c_{0,0}$ substantially bigger than a typical nuclear radius were filtered out. The result of this filter in seen in the following figure S3.

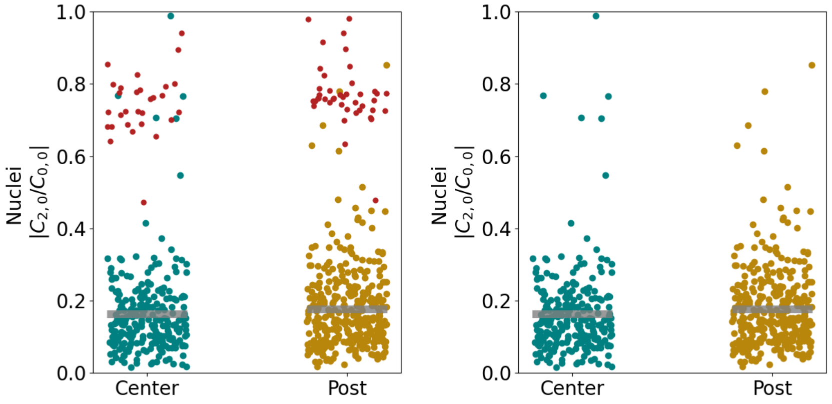


Figure S4: Deformation parameter of nuclei in the Center and Post regions of a reconstituted muscle tissue. An exploration of the segmentation results shows multi-nuclear detections, a typical issue in muscle tissue where fused cells exhibit chains of nuclei in very close proximity. Filtering detections more than twice the size of a typical nucleus ($c_{0,0} > 25$µm, red dots in the left figure) after the segmentation is performed results in the clean dataset (right figure).

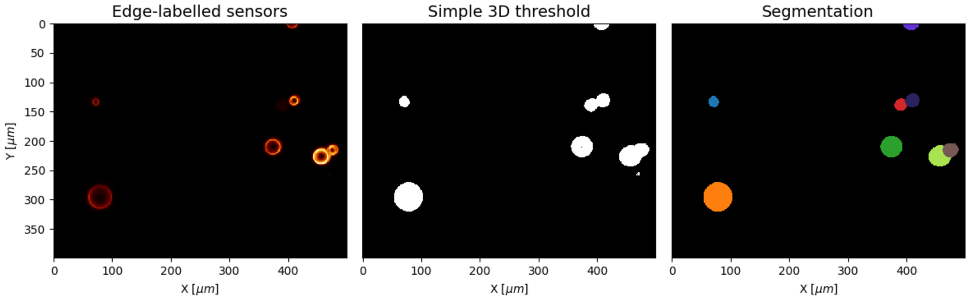


Figure S5: Edge-labelled structures can be detected in BeadBuddy with some pre-processing. In this case, a simple intensity threshold helps separating the beads from the dark background, before analyzing the data with our segmenter. A dedicated edge-segmenter will be implemented in the next iteration of BeadBuddy to directly address this possibility without treatment of the microscopy data.
